# Biomarker affliction classes contribute additively to observed dementia severity and prospective conversion risk

**DOI:** 10.1177/13872877261458678

**Published:** 2026-06-24

**Authors:** Donald R. Royall, Raymond F. Palmer, for the Alzheimer's Disease Neuroimaging Initiative

**Affiliations:** 1 Department of Psychiatry, 14742 the University of Texas Health Science Center, San Antonio, TX, USA; 2 Glenn Biggs Institute for Alzheimer's and Neurodegenerative Disorders, San Antonio, TX, USA; 3 Department of Family and Community Medicine, 14742 the University of Texas Health Science Center, San Antonio, TX, USA

**Keywords:** aging, Alzheimer's disease, amyloid, biomarkers, cognition, dementia, neurodegeneration

## Abstract

**Background:**

Dementia is likely to be overdetermined by the independent contributions of its biomarkers, of which Alzheimer's disease (AD)-specific biomarkers are a subset.

**Objective:**

To assess the impact of affliction by multiple biomarkers on dementia severity.

**Methods:**

Using previously validated algorithms, N = 988 PET (+) subjects of the Alzheimer's Disease Neuroimaging Initiative (ADNI) were assigned to groups “afflicted by” or “resilient against” the effects of central nervous system amyloid-β (Aβ), plasma adipokines, and/or neurodegeneration and tested against Clinical Dementia Rating Scale-Sum of Boxes (CDR-SB) and time to dementia conversion.

**Results:**

CDR-SB rose as a function of the number of afflicting biomarkers in both demented and non-demented cases. 327/988 (33.1%) were afflicted by a single biomarker. That biomarker was Aβ in only 19.88% of these PET (+) subjects. 221/988 (22.4%) were afflicted by all three. Only 124/988 (12.6%) were resilient to all three. Affliction by adipokines had the strongest effect (r = 0.33, p < 0.001). Affliction by Aβ was weakest (r = 0.18, p < 0.001). The number of afflicting biomarkers explained 25.2% of CDR variance and significantly impacted conversion risk over 48 months (by χ2 (df = 3) = 56.69, p < 0.001) independently of baseline CDR-SB (by Cox Proportional Hazards Wald χ2 (df = 3) = 26.24, p < 0.001).

**Conclusions:**

The biomarkers that determine dementia severity in PET (+) subjects may ultimately comprise *ad hoc* combinations and do not necessarily include Aβ. These findings have implications for A/T/N diagnoses.

## Introduction

The syndrome of dementia is likely to be overdetermined, i.e., by the summed effects of multiple competing and relatively independent dementia-related processes. Not only is there wide variability in the pattern and location of Alzheimer's disease (AD) pathology,^1^ multiple neurodegenerative pathologies are often found together both in demented and non-demented persons.^2^ In a cohort of 6262 subjects from the National Alzheimer's Coordinating Center (NACC), Maldonado-Diaz et al.^3^ report that AD neuropathologic change, Lewy body disease, age and human transactive responsive DNA-binding protein (TDP)-43-related limbic-predominant neuropathologic change, cerebrovascular disease, hippocampal sclerosis, Pick disease, and frontotemporal lobar degeneration with TDP-43 made independent contributions to overall cognitive performance. 95.7% of subjects had at least one neurodegenerative finding at autopsy. 75.5% had at least two. Other risk factors, such as age, the apolipoprotein (*APOE*) ε4 allele, and depression^4^ contribute to variation in dementia severity independently of neurodegenerative pathology (-ies).

It is widely presumed that observed pathology, its biomarker proxies, and other risk factors are 1) responsible for each individual's observed dementia severity and 2) are indicators of a single overarching diagnostic phenotype, e.g., “AD”. These assumptions are rarely tested empirically and may not be valid. About one-third of older individuals have a pathologic diagnosis of intermediate or high AD neuropathologic changes at autopsy despite normal cognition proximate to death.^5^

We have been using latent variables derived from structural equation models (SEM) to refine dementia's assessment. By this approach, we can empirically estimate the degree of functionally-salient cognitive change across the full spectrum of dementia severity,^6–7^ ascertain the biomarkers associated with dementia severity,^8–9^ including those that mediate the effects of dementia-related risk factors,^10^ and identify subsets of individuals who are “afflicted” (i.e., their dementia severity is made worse) by versus “resilient” (i.e., their dementia severity is unaffected or improved) against the unique effects of individual biomarkers and risk factors.^11–12^

We have often observed that biomarker effects act independently of each other and are individually responsible for only small fractions of observed dementia severity.^13^ It is by their aggregate effects that a substantial fraction of dementia's variance can be explained. Individuals who are largely demented by a single biomarker or risk factor are rare.^14^ This suggests that intervention on multiple biomarkers and risk factors will be required to achieve desirable outcomes and such a personalized approach will necessitate the recognition of affliction by multiple biomarkers or risk factors *in individuals*.

We have made considerable progress towards that goal. Using confirmatory bifactor models, we can isolate the variance in cognitive performance related specifically to impairments in Instrumental Activities of Daily Living (IADL). The resulting construct, i.e., “δ” (for dementia), offers a *dementia-specific* psychometric phenotype. It achieves high areas under the receiver operating characteristic curves for distinguishing all cause dementia from non-demented states, i.e., normal cognition (NC) and mild cognitive impairment (MCI),^15^ but cannot distinguish between any two dementing conditions.^16^ This implies first that all dementias share a common vector of disablement (i.e., δ) on which they might be equated and second that the cognitive changes *that distinguish* the various dementias *are not functionally salient*. δ can be reified as a factor composite and assigned to individuals as a “d-score”. Because d-scores are continuously distributed, δ effectively converts “dementia” from a category to a dimension.

As dementia's essential feature, all dementia-related biomarkers are likely to be biomarkers of δ. δ is associated with dementia-related demographic features, including age and depression,^10,17^ the *APOE* ε4 allele,^18^ pro- and anti-inflammatory blood-based protein biomarkers,^9,19^ AD neuropathology,^20^ certain “AD-specific” cerebrospinal fluid (CSF) biomarkers,^8^ central nervous system (CNS) amyloid-β (Aβ) by positron emission tomography (PET), i.e., using Amyvid florbetapir; formerly AV45,^21^ and neurodegeneration.^22^

We have found there to be interindividual variability with regard to the clinical impact of δ-related biomarkers. By a novel Line of Identity (LOI) algorithm, we have demonstrated subgroups that are “resilient” against versus “afflicted” by adipokines,^12^ inflammatory cytokines,^11^ Aβ PET,^21^ and neurodegeneration.^22^ Detailed descriptions of our algorithm are presented in those references.

In each case, LOI class assignment explains variance in dementia severity independently of covaried biomarkers and risk factors and predicts prospective conversion from non-demented states. These analyses also confirm that affliction class is uniquely determined by the biomarker of interest, and not by other δ-related biomarkers, and that class membership moderates the biomarker's association with δ, thereby meeting a strong statistical definition of “resilience” according to widely accepted criteria.^23^

Three LOI classifiers were included in this analysis, all of which were derived from the dTEL homolog.^12,21,22^ Our previously reported inflammation LOI classifier was not used as it was based on a different δ homolog.^13^

The adipokine classifier (ADPLOI) was developed to assess the impact of seven plasma adipokines on δ.^12^ N = 704/1737 = (40.53%) of ADNI participants were found to be afflicted by adipokines. Class assignment moderated the association between adipokines and CDR-SB, and afflicted subjects progressed to clinical “AD” at a faster pace than resilient cases.

The neurodegeneration classifier (NLOI) was developed to assess the impact of neurodegeneration on δ.^22^ Neurodegeneration was assessed as a latent variable indicated by magnetic resonance imaging (MRI) PET and CSF biomarkers. N = 857/1737 (49.40%) of ADNI participants were found to be afflicted by neurodegeneration. Class assignment moderated the association between neurodegeneration and CDR-SB, and afflicted subjects progressed to clinical “AD” at a faster pace than resilient cases.

The amyloid PET classifier (ALOI) was developed to assess the impact of CNS Aβ on δ.^21^ Aβ was assessed by AV45 PET regional standardized uptake values (SUVr). The association of Aβ with CDR-SB was moderated by the presence or absence of a “positive” PET result (SUVr>1.2). As there was no association below that threshold, LOI class assignment was made only in PET (+) subjects (N = 988). Afflicted Aβ PET (+) subjects (N = 388/988) progressed to clinical AD at a faster pace than resilient cases.

For this analysis, we hypothesize that LOI-assigned affliction classes will contribute independently to dementia severity, as estimated by the Clinical Dementia Rating Scale (CDR)^24^ Sum of Boxes (CDR-SB),^25^ and also to the prospective risk of conversion to clinical “AD” from non-demented states. We further predict that the number of affliction classes to which an individual is assigned will contribute to dementia severity, and prospective conversion risk. If so, this will support the conceptualization of “AD” as an overdetermined syndrome, with far ranging implications for dementia's assessment, treatment and the ATN(X) project.^26^

## Methods

Briefly, this is a secondary analysis of data from the Alzheimer's Disease Neuroimaging Initiative (ADNI, adni.loni.usc.edu).^27^ The ADNI was launched in 2003 as a public-private partnership, led by Principal Investigator Michael W. Weiner, MD. The original goal of ADNI was to test whether serial MRI, PET, other biological markers, and clinical and neuropsychological assessment can be combined to measure the progression of MCI and early AD. The current goals include validating biomarkers for clinical trials, improving the generalizability of ADNI data by increasing diversity in the participant cohort, and to provide data concerning the diagnosis and progression of AD to the scientific community. For up-to-date information, see adni.loni.usc.edu. The subjects and methods have been described elsewhere.^11,20–21^ LOI class membership for adipokines (ADPLOI),^12^ Aβ PET (ALOI),^21^ and neurodegeneration (NLOI)^22^ has been previously assigned relative to dementia severity as measured by the “dTEL” δ homolog.^28^ dTEL's construction, and its parameter weights in these subjects, are provided in Supplemental Figure 1. The biomarkers were assessed as in their affliction classes’ original validations. The impact of affliction class on clinical dementia was assessed relative to CDR-SB.^25^ Higher scores are more impaired. Clinical diagnosis (DX) was categorized, i.e., NC = 0, MCI = 1, AD = 2.

dTEL has been engineered to facilitate telephone administration, although its indicators were not administered remotely in ADNI. It is indicated by Logical Memory I (LMI) and II (LMII) from the Wechsler Memory Scale^29^ and Category Fluency (Animals) from the Consortium to Establish a Registry for Alzheimer's Disease (CERAD) battery.^30^ dTEL's IADL indicator in ADNI is the Functional Assessment Questionnaire (FAQ).^31^

### Statistical analyses

#### Approach

Our LOI algorithm has been previously described.^12–13,21–22^ Briefly, a δ homolog is regressed in SEM onto a biomarker of interest. This creates a residual, whose variance is captured as a new latent variable, i.e., the cognitive residual (CR). The SEM model is presented in Supplemental Figure 2.

CR is arguably a measure of cognitive reserve.^21^ It represents the variance in dementia severity attributable to any and all influences excepting the biomarker of interest. When standardized CR scores are correlated with unadjusted d-scores (which are also standardized), a line of identity can be drawn through the resulting scatterplot. Each subject can then be assigned to “Afflicted” versus “Resilient” classes by their position in the scatterplot relative to the LOI.

Alternatively, a continuous difference score can be calculated as the difference between unadjusted d-scores and CR. A difference score of “zero” corresponds to an individual's placement on the LOI. Difference scores > or < 0 indicate affliction by, or resilience against, the specific effect of the biomarker of interest (with class assignment being determined by the polarity of the δ homolog's association with dementia severity). Each difference score is specific to the effect of the biomarker used as the independent variable in Supplemental Figure 2.

Supplemental Figure 3 presents a histogram of difference scores relating to the effect of the Aβ PET SUVr on dTEL. Because dTEL and CR are both positively associated with CDR-SB, higher scores are more adverse. At the LOI, dTEL = CR and the difference score is zero, i.e., Aβ has made no impact on δ, despite a positive scan result (in this sample). When CR scores fall below the LOI, dementia severity has improved after Aβ's impact is adjusted. Such cases were being afflicted by Aβ and will present with a positive difference score. Conversely, if CR rises above the LOI, dementia severity has worsened after Aβ's effect is adjusted. Aβ then, was arguably *protecting* those participants, despite their positive PET scans. They are resilient against Aβ's putative adverse effect. Such cases will have a negative difference score.

Our LOI algorithm purports to isolate the impact of a single biomarker of interest on δ. If so, then each affliction class should impact CDR-SB independently of the others, excepting possible interactions or synergistic effects. Affliction by multiple biomarkers may be summative, or synergistic. As these biomarkers vary in regard to the strengths of their associations with δ, the impact of affliction may vary with the specific biomarker(s) in question.

To address these issues, we constructed a dummy variable, “ASUM”, representing the number of affliction classes to which each subject belonged. ASUM scores ranged from 0 to 3. An ASUM score of 0 indicated that a subject is resilient against each targeted biomarker. A score of 3 indicates affliction by all three. Of course, these three are merely a subset of all possible biomarkers that may be contributing to the CDR-SB. So, an individual might also be afflicted by yet other unmodeled determinants.

The independent effects of each affliction class on CDR-SB were tested by multivariate regression. The effect of affliction by multiple biomarkers was assessed by time to AD conversion from non-demented baseline diagnosis (i.e., NC or MCI). Time to initial AD conversion was calculated, and the effect tested by survival analysis using ASUM scores as the predictor (i.e., by Kaplan-Meier curves for multiple groups). The independent effects of ASUM and each specific affliction class on conversion risk was assessed by Cox proportional hazards models using baseline CDR-SB as a covariate.^32^

## Results

**Figure 1.**
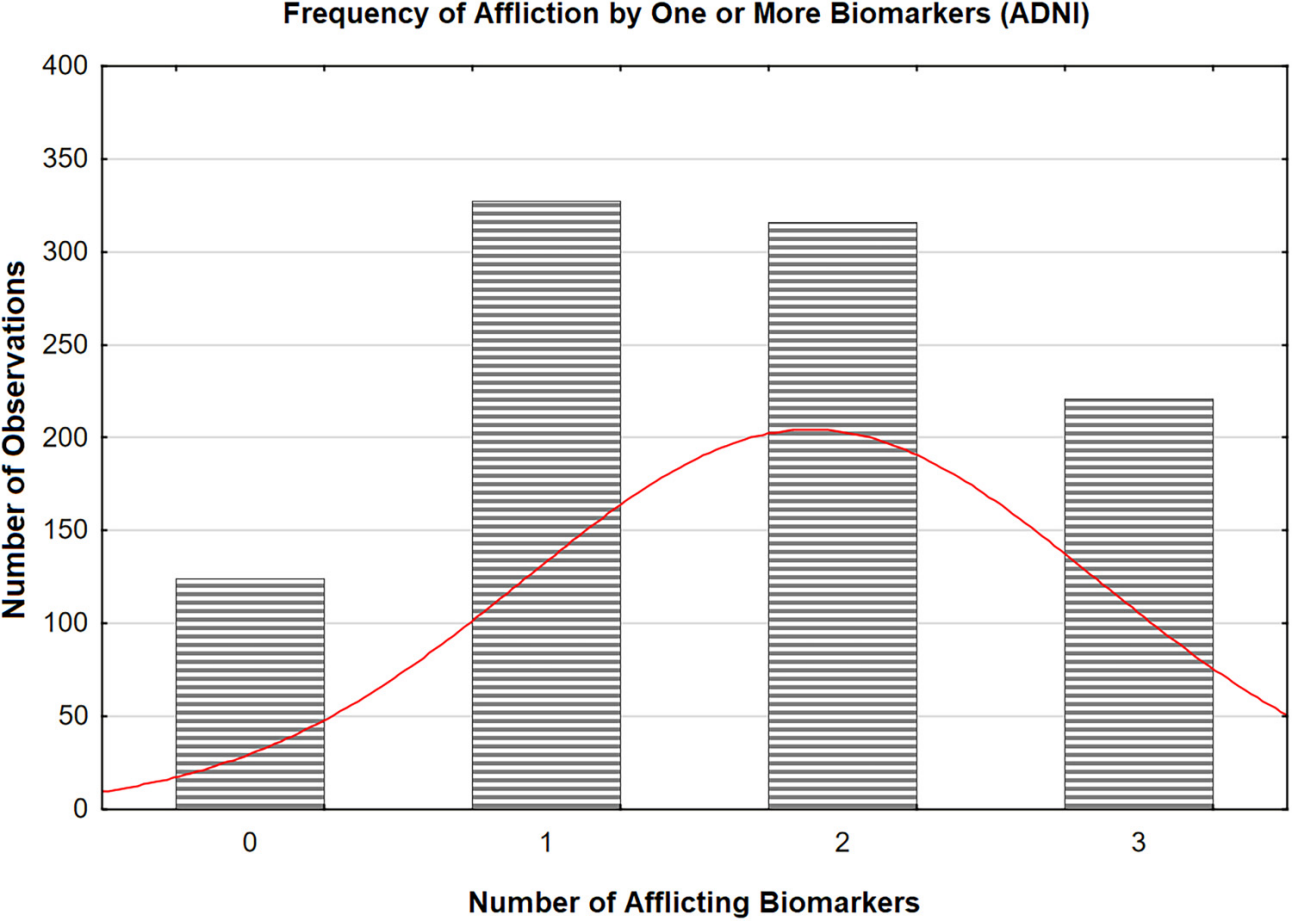
Frequency of affliction by one or more biomarkers in PET (+) participants (ADNI).

ADNI: Alzheimer's Disease Neuroimaging Initiative; PET: positron emission tomography.

Table 1 presents descriptive statistics for the entire sample and by the number of afflicting biomarkers. The majority of subjects were afflicted by one or more of the modeled biomarkers. Only 124/988 (12.6%) were resilient to all three. 327/988 (33.1%) were afflicted by a single biomarker. That biomarker was Aβ in only 19.88% of these PET (+) subjects. 221/988 (22.4%) were afflicted by all three. A histogram of ASUM scores is presented in Figure 1.

**Table 1.** Descriptive statistics by the number of afflicting biomarkers.^a^

Variable	All cases N = 988 Mean (SD)	Afflicting biomarkers 0 N = 124 /12.6%	Afflicting biomarkers 1 N = 327 /33.1%	Afflicting biomarkers 2 N = 316 / 32.0%	Afflicting biomarkers 3 N = 221 /22.4%
Age (years)	74.68 (7.28)	72.41 (7.30)^b^	74.73 (6.88)	75.45 (7.23)	74.76 (7.71)
Gender (%♂)	57.09	68.55^d^	62.69^d^	54.11	46.61
Ethnicity (% Hispanic)	3.18	2.44	3.09	1.60^b^	5.91
*APOE* ε4 allele (%)	77.32	58.07^d^	76.00	78.59	88.48
AV45 SUVr	1.37 (0.12)	1.27 (0.05)^d^	1.33 (0.11)^d^	1.36 (0.11)^d^	1.49 (0.10)
CDR-SB	2.39 (1.85)	1.27 (1.18)^d^	1.59 (1.27)^d^	2.63 (1.72)^d^	3.88 (1.99)
EDUC (years)	15.43 (3.00)	15.57 (2.91)	15.82 (2.99)^c^	15.34 (2.92)	14.93 (3.13)
MMSE	26.01 (2.69)	27.66 (2.04)^d^	27.18 (1.92)^d^	25.75 (2.49)^d^	23.70 (2.58)
Afflicted by Aβ (%)	39.27	0.00^d^	19.88^d^	32.28^d^	100.00
Afflicted by Adipokines (%)	58.60	0.00^d^	30.28^d^	81.96^d^	100.00
Afflicted by neurodegeneration (%)	66.30	0.00^d^	49.85^d^	85.76^d^	100.00
ASUM	1.64 (0.96)	0	1	2	3

All subjects are Aβ PET (+).

^a^ ASUM is significantly associated with all variables excepting ethnicity by ANOVA, all p ≤ 0.001.ADNI: Alzheimer's Disease Neuroimaging Initiative.

^b^ differs from ASUM = 3 by Tukey's HSD, p < 0.05.

^c^ differs from ASUM = 3 by Tukey's HSD, p < 0.01.

^d^ differs from ASUM = 3 by Tukey's HSD, p < 0.001.

Aβ: amyloid-beta by PET; CDR-SB: Clinical Dementia Rating Scale-Sum of Boxes; EDUC: education; MMSE: Mini-Mental State Exam; SD: standard deviation; PET: positron emission tomography; SUVr: regional standardized uptake values.

**Figure 2.**
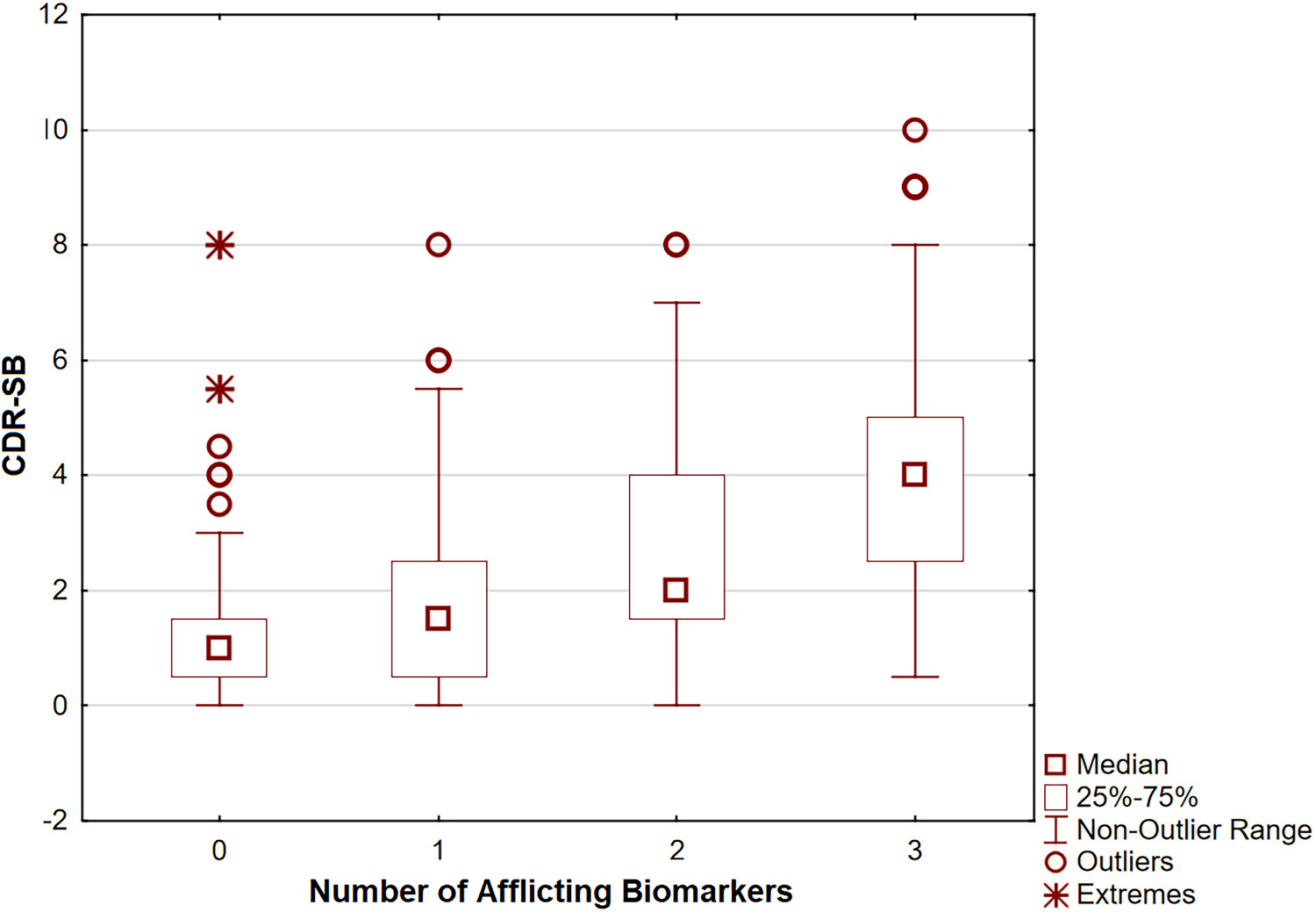
CDR scores rise as a function of the number of afflicting biomarkers in PET (+) participants (ADNI).

ADNI: Alzheimer's Disease Neuroimaging Initiative; CDR: Clinical Dementia Rating scale; CDR-SB: CDR-Sum of Boxes; PET: positron emission tomography. Higher scores are more impaired.

**Figure 3.**
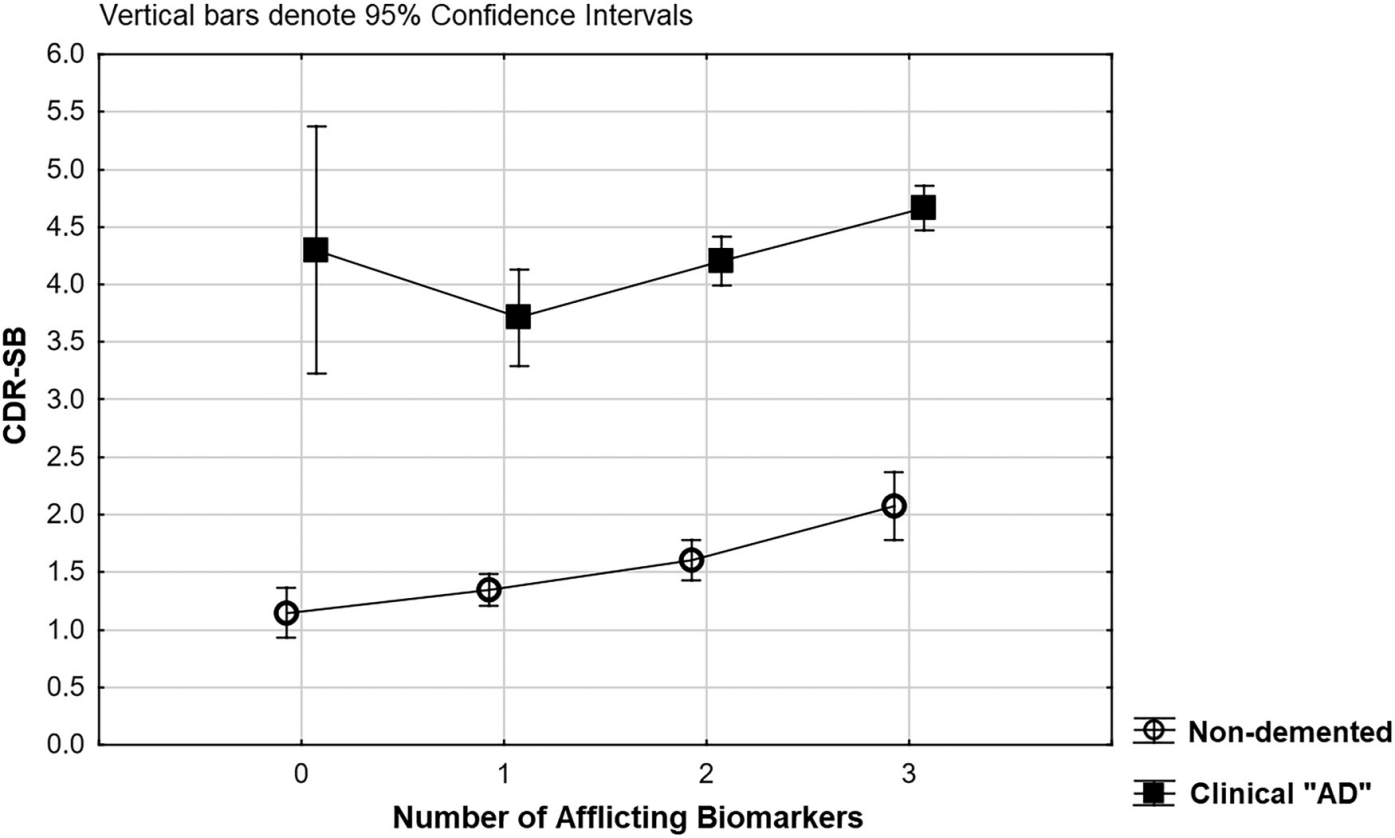
No interaction between dementia diagnosis and the number of afflicting biomarkers. Biomarkers impact CDR-SB in both demented and non-demented PET (+) participants by factorial ANOVA: F (93, 980) = 0.66, p = 0.57 (ADNI)].

ADNI: Alzheimer's Disease Neuroimaging Initiative; AD: Alzheimer's disease; ANOVA: analysis of variance; CDR-SB: Clinical Dementia Rating scale-Sum of Boxes; PET: positron emission tomography. Higher scores are more impaired.

The number of afflicting biomarkers was significantly associated with all the variables in Table 1 excepting ethnicity (by ANOVA, all p ≤ 0.001). The ethnic diversity in ADNI's sample is very low. CDR-SB rose as a function of the number of afflicting biomarkers (Figure 2) and in both demented and non-demented cases (Figure 3). However, there was no interaction between clinical diagnosis and the number of afflicting biomarkers (Figure 3).

Each affliction class contributed independently to the CDR-SB (Table 2). Affliction by adipokines had the strongest effect (β = 0.33, p < 0.001). ALOI explained the least variance in dementia severity (β = 0.18, p < 0.001). Together, affliction by these biomarkers explained 25.2% of the variance in CDR-SB ratings.

**Table 2.** Affliction classes contribute independently to CDR-SB.

N = 988	β	SE of β	b	SE of β	t(984)	p
Intercept			0.80	0.10	7.82	<0.001
ALOI	0.18	0.03	0.67	0.11	6.32	<0.001
ADPLOI	0.33	0.03	1.23	0.11	11.52	<0.001
NLOI	0.23	0.03	0.92	0.11	8.24	<0.001

R^2^ = 0.25; F (3984) = 111.75, p < 0.001.

ALOI: amyloidopathy class; ADPLOI: adipokines affliction class; β: standardized estimates; CDR-SB: Clinical Dementia Rating Scale-Sum of Boxes; NLOI: neurodegeneration affliction class; SE: standard error.

Biomarker affliction is understandably strongly associated with the total number of afflicting biomarkers but only moderately with clinical diagnosis (Table 3). This is consistent with the relatively small fraction of CDR-SB variance explained by the three affliction classes. Interestingly, affliction by Aβ, favors affliction by both adipokines and neurodegeneration. The converse is not true. Given affliction by either of the latter two, only a minority were co-afflicted by Aβ (in these PET (+) cases!) (Table 3).

**Table 3.** Biomarker affliction contributes strongly to the total number of afflicting biomarkers and moderately with clinical diagnosis. Affliction by Aβ favors affliction by adipokines and neurodegeneration. The converse is not true.

Variable	Frequency (%) of comorbid affliction (Left three columns) given affliction by one (Far left). Spearman Rank Order Correlations (Right two columns).				
	ALOI	ADPLOI	NLOI	ASUM	DX
ALOI	100	68.56	71.65	0.630^a^	0.260^a^
ADPLOI	37.78	100	68.47	0.708^a^	0.457^a^
NLOI	26.45	45.86	100	0.645^a^	0.299^a^
ASUM				1.000	0.519^a^
DX				0.519^a^	1.000

^a^ p < 0.001.

ALOI: Aβ affliction class; ADPLOI: adipokine affliction class; ASUM: total number of afflicting biomarkers; DX: categorized diagnosis; NLOI: neurodegeneration affliction class.

**Figure 4.**
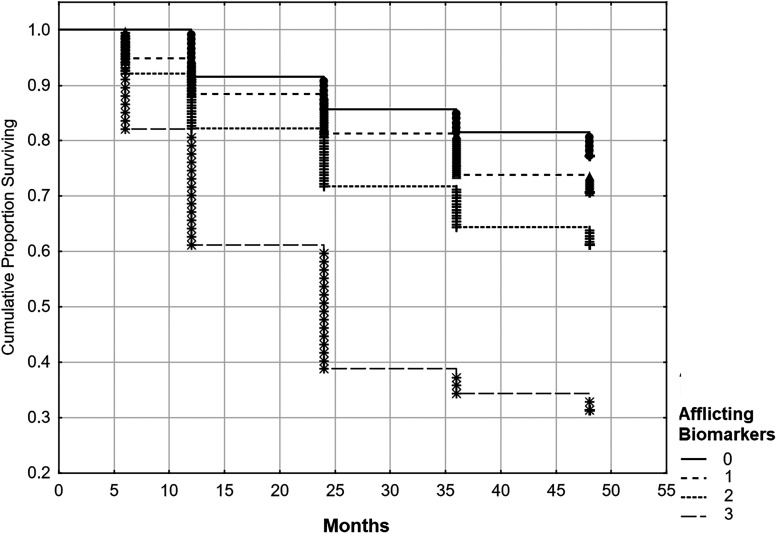
Time to conversion as a function of the number of afflicting biomarkers in PET (+) participants (ADNI).

ADNI: Alzheimer's Disease Neuroimaging Initiative; PET: positron emission tomography.

Figure 4 presents survival curves for dementia conversion as a function of the number of afflicting biomarkers. Given affliction by none, including Aβ in these PET (+) subjects (N = 119), there is still a 20% conversion risk by 48 months. Their conversion risk likely reflects the impact(s) of unmodeled risk factors. Membership in any single affliction class (N = 294) had no incremental effect above membership in none (by Gehan's Wilcoxon test = 1.49, p = 0.14). Membership in any two affliction classes (N = 191) significantly increased conversion risk relative to membership in only one (by Gehan's Wilcoxon test = 2.36, p = 0.02). The risk is significantly increased again if a third afflicting biomarker is added (by Gehan's Wilcoxon test = 4.56, p < 0.001) (N = 67).

All three affliction classes are significantly associated with CDR-SB (Table 2) and with clinical diagnosis (Table 3). Regardless, the number of afflicting biomarkers added to conversion risk independently of baseline CDR-SB (by Cox Proportional Hazards Wald χ^2^ (df 3) = 26.24, p < 0.001).

### Caveat

One limitation of our approach is that resolving LOI-derived differences to dichotomous classifiers reduces the information available relative to the continuous difference score. For example, we reported in Table 2 that biomarker LOI classes explained 25.2% of the variance in CDR-SB ratings. 66.5% can be explained if difference scores are used (Supplemental Table 1), i.e., more than the observed biomarkers themselves (R^2^ = 46.3%, Supplemental Table 2).

## Discussion

Our findings extend earlier findings from both ADNI and the Texas Alzheimer's Research and Care Consortium (TARCC). The LOI algorithm generates biomarker-specific classifiers that independently contribute variance to observed ratings of dementia severity and sum to prospectively predict dementia conversion from non-demented baselines. These findings touch upon several important unresolved issues in the literature. Dementia is likely to be over determined. Clinicians risk overestimating the significance of “positive” biomarker findings by conflating risks attributable to the biomarker of interest with unmodeled and competing biomarker effects. Summative effects may be more explanatory, but independent effects would undermine the coherence of clinical diagnoses. Diagnostic schema, such as the A/T/N (X) system, may be misleading unless more attention is paid to covaried effects. The possibility of resilience to some biomarker effects is a further complication, but offers opportunities to improve outcomes by targeting afflicted cases, and modulating the determinants of resilience.

### Dementia is overdetermined

These findings support the conceptualization of dementia as an overdetermined syndrome. Each LOI class contributed independently to dementia severity and individuals varied as a function of both the number and combination of affliction classes to which they belonged. Moreover, few demented subjects were afflicted by only one of the modeled biomarkers and even in those cases, affliction by unmodeled biomarkers cannot be dismissed. The majority of subjects afflicted by a single biomarker were without dementia at baseline. Their near-term conversion risk was barely distinguishable from those belonging to no affliction class and significantly lower than those belonging to more than one. That suggests that the demonstration of any one of these biomarkers in a demented patient may not provide a sufficient explanation for their dementia.

It is more likely that these biomarkers erode cognitive “reserve” yet remain unable to effect conversion on their own. Some subjects with dementia were resilient to all three modeled biomarkers (Figure 1). Their dementias presumably resulted from the effects of unmodeled δ-related biomarkers and /or risk factors. We have yet to construct a classifier for tauopathy and expect some of the unexplained variance will be attributable to its impact.

Figure 1 shows that a minority of Aβ PET (+) ADNI subjects are afflicted by all three modeled biomarkers, and Figure 3 shows that not all such cases are demented. Multiply afflicted cases at lower CDR-SB scores are not resilient to the biomarkers of interest here. Their membership in the modeled afflicted classes confirms the adverse impact of those biomarkers on their d-scores. It is more likely that they retain adequate levels of cognitive reserve, attributable to unmodeled processes and biomarkers. They may be afflicted by fewer *unmodeled processes* relative to their demented brethren. Alternatively, they might be resilient to them. Regardless, affliction by all three of the modeled biomarkers will necessarily have eroded those reserves, consistent with accelerated times to conversion in Figure 4.

### Prospective conversion

It remains to be determined whether the dose-related risk of prospective dementia conversion (Figure 4) is explained by progression of known afflicting biomarkers, or recruitment and /or progression of unmodeled conditions. Longitudinal analyses have described multiple sequences of A/T/N biomarker status conversions from an initially A-,T-,N- state,^33^ but the association between those biomarker changes and δ is not known. Some studies have associated incident A/T/N state conversions to domain-specific cognitive changes,^34^ but observed cognitive performance is only partly explained by δ and any residual variation has little impact on functional abilities.^35^ We can now assess the longitudinal changes in affliction class membership and the factors or biomarkers associated with those transitions.

### Resilience

It is remarkable that although all these subjects were PET (+) for Aβ, the majority of those afflicted by a single biomarker were not afflicted by Aβ. This can be partially explained by the relatively high frequency of comorbid afflictions among those afflicted by that biomarker (Table 3). Regardless, only a minority of these PET (+) cases were multiply afflicted by the biomarkers under study. We previously showed that <40% of PET (+) ADNI participants are afflicted by Aβ and that the majority of the afflicted subset were not clinically demented.^21^ The low frequency of affliction by Aβ among PET (+) cases may reflect resilience to that biomarker instead.

Our analyses have not addressed the mechanisms of resilience against any of these biomarkers. We know not whether resilience is achieved by processes that are unique to each biomarker, or common across several. Nor is it obvious that “resilience” translates into protection from the adverse effects of a biomarker. Previous studies show that affliction class moderates a biomarker's effect. The associations of inflammatory cytokines,^11^ adipokines,^12^ and neurodegeneration,^22^ with dementia are attenuated in their resilient classes, but still adverse. On the other hand, Aβ PET has no association with dementia in cases resilient to its effect.^21^

Regardless, this approach offers a way forward. We can examine either cross-sectional or longitudinal cross-class differences for clues as to the mechanisms involved. We can look at the stability of class membership over time and across stages of dementia's evolution and at the biomarkers associated with those transitions. We can search for communalities in resilience across several biomarkers.

### Relevance to A/T/N

The A /T/ N (X) scheme provides for a biomarker-based diagnosis of “AD”.^25^ Cases are classified *in vivo* by biomarkers purported to be indicative of the hallmark pathological features of AD, i.e., amyloidopathy (A), tauopathy (T), and neurodegeneration (N). “X” allows for other AD-related biomarkers, both known and to be discovered. These current findings have relevance to this project.

First, the mere presence of a biomarker does not imply affliction by it. Recall that about one-third of older individuals have a pathologic diagnosis of intermediate or high AD neuropathologic changes at autopsy despite normal cognition proximate to death.^5^ We found affliction by multiple biomarkers explained a minority of the variance CDR-SB. The remainder may be explained by other unmodeled biomarkers and risk factors. So, confirmation of A/T/N biomarkers in a patient may not fully explain their dementia severity, and correcting A/T/N impacts may leave other afflicting conditions unaddressed.

It also should be recalled that “zero afflictions” does not necessarily mean that a case lacks the biomarkers of interest. In the context of A/T/N ratings, the fraction with no afflictions might not be A-/T-/N-. Instead, they might be A+/T+/N + . These subjects were PET (+) for Aβ, but <40% were afflicted by it.^22^ Subjects who are members of zero affliction classes are resilient to all three regardless of their observed levels.

Resilience may explain the disheartening clinical response to otherwise successful biomarker reductions and undermines the rationale for proposals to approve interventions on the basis of their biomarker effects rather than their clinical efficacy. Past interventions have been made on unselected subjects, many of whom might have been resilient to the targeted biomarkers. This may help explain notoriously failed trials of anti-inflammatory and Aβ interventions.^36–37^ Those interventions might have been more successful had they been directed to an afflicted class of subjects. Affliction status cannot be determined by biomarkers alone.

This suggests A/T/N (X) classification should be modified to address resilience. However, that could quickly become unwieldy. Simply qualifying A, T and N effects into afflicted and resilient subsets would expand the number of A/T/N classes from eight currently, to 27. Moreover, A/T/N is not a comprehensive description of dementia's biomarkers.

Current A/T/N nomenclature cannot handle this complexity well. As these are all PET (+) cases they would be rated as A+, even though the majority are not afflicted by Aβ. Any case with evidence of neurodegeneration, would be rated A + N+, even though only a faction are afflicted by that finding as well. So, depending on LOI findings, such cases might belong to 0, 1 or 2 biomarker affliction classes, with significant differences in their clinical prospects (Figure 4). Moreover, this analysis did not consider tauopathy, adding more complexity.

Adipokines are associated with tauopathy^38^ and afflicted higher fractions of these Aβ PET (+) cases than did Aβ. They could be addressed by “X” in the A/T/N (X) scheme. *APOE* and depression contribute to dementia severity independently of all these effects. Some patients may be resilient to their effects as well. A/T/N alone does not offer a comprehensive assessment of these dementia risks

### Relevance to “AD”

A/T/N's weaknesses belie misconceptions of AD and its association with dementia. The A/T/N scheme was originally proposed as an aid to the diagnosis of AD, not dementia. The current analysis shows that it may prove inadequate to the latter task. It considers only a subset of δ-related biomarkers and ignores resilience against A/T/N biomarker effects. If we agree that A/T/N biomarkers are essential to AD, we will need to acknowledge 1) that AD explains only a fraction of the dementia in a given case, even if A + T + N+, and 2) that intervention on A/T or N may result in only partial improvements in dementia severity. Still other, unconsidered, determinants of the d-score remain to be acted on and might perhaps be more remediable. If we still want to diagnose AD by ATN, we may need to distinguish those biomarkers’ covaried from their independent effects. Would A+, T + and N + pathology still indicate “AD” if one or more of those domains had no association with the others and co-occurred randomly in some subset of patients?^39–40^

Table 3 offers limited support for the validity of AD's diagnosis by summed biomarker effects. Affliction by Aβ, arguably the most AD-specific of our modeled biomarkers, selects for affliction by others. We reported 49.40% of ADNI participants to be afflicted by neurodegeneration^19^ but 71.65% of Aβ afflicted cases are so-affected (Table 3). That suggests that these biomarkers are covaried. Conversely, affliction by adipokines or neurodegeneration, arguably less-AD specific conditions, did not select for affliction by Aβ, even in these PET (+) cases. There are similar reports of N/T mismatch.^39^ In fact, the relatively low rates of Aβ affliction in those conditions could be interpreted as a source of resilience against Aβ, as these are all PET (+) cases. 39.3% of ADNI participants with (+) PET findings were found to be afflicted by Aβ^17^ versus 26.45% of those afflicted by neurodegeneration (Table 3).

A more satisfactory approach might be to construct a latent variable using A, T and N biomarkers as indicators. Such a construct would represent an AD specific systems level phenotype. It could then be submitted to LOI and affliction (or resilience to it) diagnosed. That would offer a practical approach to the diagnosis of AD as a functionally-salient disorder characterized by covaried A/T/N pathology.

### Limitations

We have found LOI-derived biomarker affliction classes to additively modulate dementia severity and prospective conversion risk. However, we did not formally assess potential interactions between individual affliction classes. It is plausible that some biomarkers may work in concert to achieve their effects while others influence dementia risk independently. This can be better assessed with a broader range of biomarkers via interaction terms or latent factor constructs.

We also admit that continuous difference scores explain more variance in dementia severity than their respective affliction classes. The difference score might then offer a better approach to the empiric testing of biomarker effects in a research context. Regardless, the use of affliction classes can facilitate the integration of resilience into clinical settings similarly to the categorization of other continuous measures. For example, dichotomized biomarker thresholds (e.g., a “positive” PET scan) which are validated against categorized clinical diagnoses and used to predict dichotomized clinical conversions in survival analyses. Likewise, dichotomous affliction classes can be used for the recruitment of subjects into clinical trials testing biomarker-specific interventions and /or for triaging cases for treatment by those interventions).

Additionally, some ADNI cases could have been misdiagnosed by clinicians. ADNI tries to overcome this issue by selecting against obvious non-AD etiologies. We have previously reported in another dataset that as many as 26% of cases with “MCI” may be misdiagnosed relative to δ.^31^ Misdiagnosed cases were more likely to be older, less well educated, or to speak English as a second language (i.e., more likely to present obstacles to clinician evaluation). One of the advantages of the d-score is that it is empirically estimable and does not depend on the skill or experience of study personnel.

Regardless, the mean CDR-SB score of multiply afflicted yet non-demented subjects in this analysis was only 2.0 at baseline (Figure 3). In their original validation of the CDR-SB, O’Bryant et al.^22^ reported a mean score of 1.3 ± 1.16 among N = 2551 MCI cases from the NACC with a range of 0–11. So, the score we observed in non-demented subjects afflicted by all three of these biomarkers is well within the range for non-demented MCI cases.

In conclusion, biomarker-specific psychometric classifiers, constructed by an LOI approach, can be used to assign individuals to affliction classes. Class membership moderates clinical outcomes in the presence of the biomarker(s) of interest. They additively explain variance in dementia severity. Few cases are demented when afflicted by a single biomarker, and some escape dementia despite affliction by several. This may be simply explained as the effects of yet other, unmodeled biomarkers. In their aggregate, our findings support the conceptualization of dementia as an overdetermined syndrome, even in the presence of disease-specific biomarkers, e.g., biomarkers of AD. The A/T/N nomenclature cannot handle this complexity well, but a latent variable approach could distinguish covaried A/T/N development from independent A/T/N effects. If such a latent A/T/N factor was submitted to this LOI analysis, individuals could be recognized as afflicted by or resilient against AD from their observed biomarkers of interest.

## Supplemental Material

sj-docx-1-alz-10.1177_13872877261458678 - Supplemental material for Biomarker affliction classes contribute additively to observed dementia severity and prospective conversion risk Supplemental material, sj-docx-1-alz-10.1177_13872877261458678 for Biomarker affliction classes contribute additively to observed dementia severity and prospective conversion risk by Donald R. Royall, Raymond F. Palmer and in Journal of Alzheimer's Disease
