# Supplementary material for: Biomarker affliction classes contribute additively to observed dementia severity and prospective conversion risk
Source: J Alzheimers Dis. 2026 Jun 24;112(3):1382–92. doi: 10.1177/13872877261458678 (PMC13392201; doi:10.1177/13872877261458678)
Supplement: sj-docx-1-alz-10.1177_13872877261458678 - Supplemental material for Biomarker affliction classes contribute additively to observed dementia severity and prospective conversion risk [file sj-docx-1-alz-10.1177_13872877261458678.docx]

**Supplemental Material**

**Biomarker affliction classes contribute additively to observed dementia severity and prospective conversion risk**

**Supplemental Table 1.** Line of Identity (LOI) difference scores have independent effects but explain a majority of CDR variance.

| **N=988** | **Dependent Variable: CDR-SB: F(3,984) = 654.93, p<0.001; R²= 0.665** | | | | | |
| --- | --- | --- | --- | --- | --- | --- |
|  | \| **b*** \| \| --- \| | \| **SE** \| \| --- \| | \| **b** \| \| --- \| | \| **SE** \| \| --- \| | \| **t(984)** \| \| --- \| | \| **p** \| \| --- \| |
| \| **Intercept** \| \| --- \| |  |  | 2.115 | 0.05 | 58.010 | <0.001 |
| \| **ADIF** \| \| --- \| | -0.057 | 0.019 | -0.382 | 0.124628 | -3.062 | 0.002 |
| \| **ADPDIF** \| \| --- \| | -0.470 | 0.041 | -0.653 | 0.057 | -11.417 | <0.001 |
| \| **NDIF** \| \| --- \| | -0.358 | 0.041 | -0.363 | 0.042 | -8.701 | <0.001 |

Aβ: amyloid-beta; ADIF: Aβ SUVr-adjusted dTEL residual - dTEL; ADPDIF: Adipokine factor-adjusted dTEL residual - dTEL; CDR-SB: Clinical Dementia Rating Scale Sum of Boxes; NDIF: NFactor-adjusted dTEL residual – dTEL; SE: standard error;

**Supplemental Table 2.** Observed biomarkers have independent effects that explain a minority of CDR variance.

| **N=988** | **Dependent Variable: CDR-SB: F(3,984) = 282.26, p<0.001; R²= 0.463** | | | | | |
| --- | --- | --- | --- | --- | --- | --- |
|  | \| **β** \| \| --- \| | \| **SE** \| \| --- \| | \| **b** \| \| --- \| | \| **SE** \| \| --- \| | \| **t(984)** \| \| --- \| | \| **p** \| \| --- \| |
| \| **Intercept** \| \| --- \| |  |  | -0.903 | 0.494 | -1.828 | 0.07 |
| \| **Aβ SUVr** \| \| --- \| | 0.131 | 0.024 | 1.979 | 0.364 | 5.441 | <0.001 |
| \| **Adipokines** \| \| --- \| | -0.270 | 0.027 | -1.077 | 0.106 | -10.120 | <0.001 |
| \| **Nfactor** \| \| --- \| | -0.463 | 0.027 | -0.867 | 0.050 | -17.269 | <0.001 |

Aβ: amyloid-beta; Adipokines: adipokine factor score; CDR-SB: Clinical Dementia Rating Scale Sum of Boxes; NFactor: neurodegeneration factor score; SE: standard error; SUVr: Standardized Uptake Value ratio.

**Supplemental Figure 1.** dTEL is defined for these analyses as a formative latent variable in structural equation modeling (SEM). It is indicated by three cognitive performance measures i.e., Logical Memory I (LMI) and II (LMII) from the Wechsler Memory Scale^1^ and Category Fluency (Animals) from the Consortium to Establish a Registry for Alzheimer’s Disease (CERAD) battery,^2^ and one measure of instrumental activities of daily living (IADL), i.e., the Functional Assessment Questionnaire (FAQ)^3^ (in ADNI). dTEL was originally developed to allow for telephone administration although, ADNI data did not involve remote administration. It is strongly associated with CDR-SB but does not depend on subjective input from experienced clinicians.


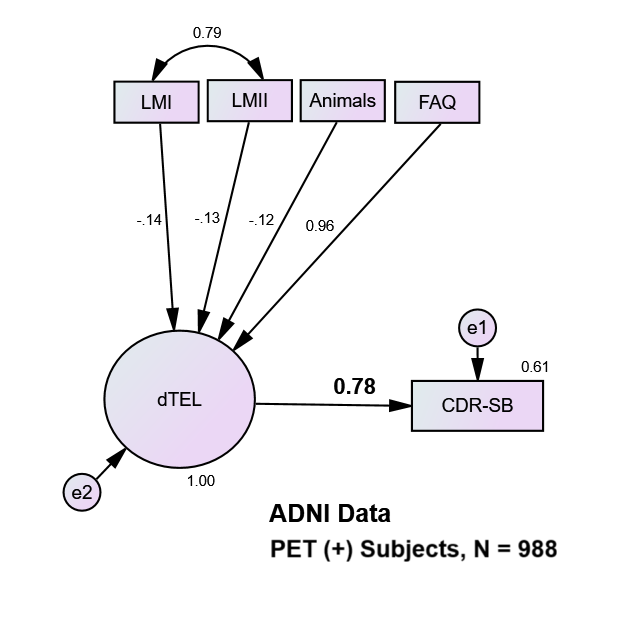


ADNI: Alzheimer’s Disease Neuroimaging Initiative; CDR-SB: Clinical Dementia Rating Scale “Sum of Boxes”. Higher scores are more impaired; LMI: Logical Memory I; LMII: Logical Memory II

**References**

1. Wechsler D. *Wechsler Memory Scale – Third Edition*. San Antonio, TX: The Psychological Corporation, 1977.

2. Morris JC, Heyman A, Mohs RC, et al. The Consortium to Establish a Registry for Alzheimer’s Disease (CERAD). Part I. Clinical and neuropsychological assessment of Alzheimer’s disease. *Neurology* 1989; 39: 1159-1165.

3. Pfeffer RI, Kurosaki TT, Harrah CH, et al. Measurement of functional activities in older adults in the community. *J Gerontol* 1982; 37: 323–329.

**Supplemental Figure 2.** CR is derived from dTEL by regression in SEM. CR by this approach would be identical to a biomarker-adjusted residual obtained via traditional regression of any dementia severity measure onto any biomarker of interest. However, the SEM model in this figure makes it explicit that CR’s variance, and hence biomarker-adjusted residuals obtained via traditional regression, is orthogonal to the effect of the biomarker. Together, these two sources of variance explain the totality of dTEl’s variance.


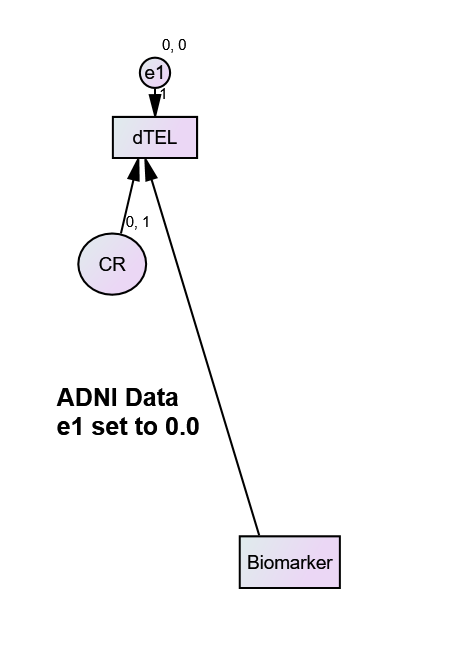


ADNI: Alzheimer’s Disease Neuroimaging Initiative; CR: cognitive residual; SEM: structural equation modeling

**Supplemental Figure 3.** Histogram of Aβ PET difference scores. Because dTEL and CR are both positively associated with CDR-SB, higher scores are more adverse. dTEL and CR differ only in the impact of the biomarker of interest. When the difference score is zero, dTEL = CR and Aβ has made no impact on δ. When CR scores fall below dTEL, dementia severity has improved after Aβ’s impact is adjusted. Such cases were being afflicted by Aβ and will present with a positive difference score. Conversely, if CR rises above dTEL, dementia severity has worsened after Aβ’s effect is adjusted. Aβ then, was arguably protecting those participants (despite positive PET scans in this sample!). They are resilient against Aβ’s putative adverse effect. Such cases will have a negative difference score.


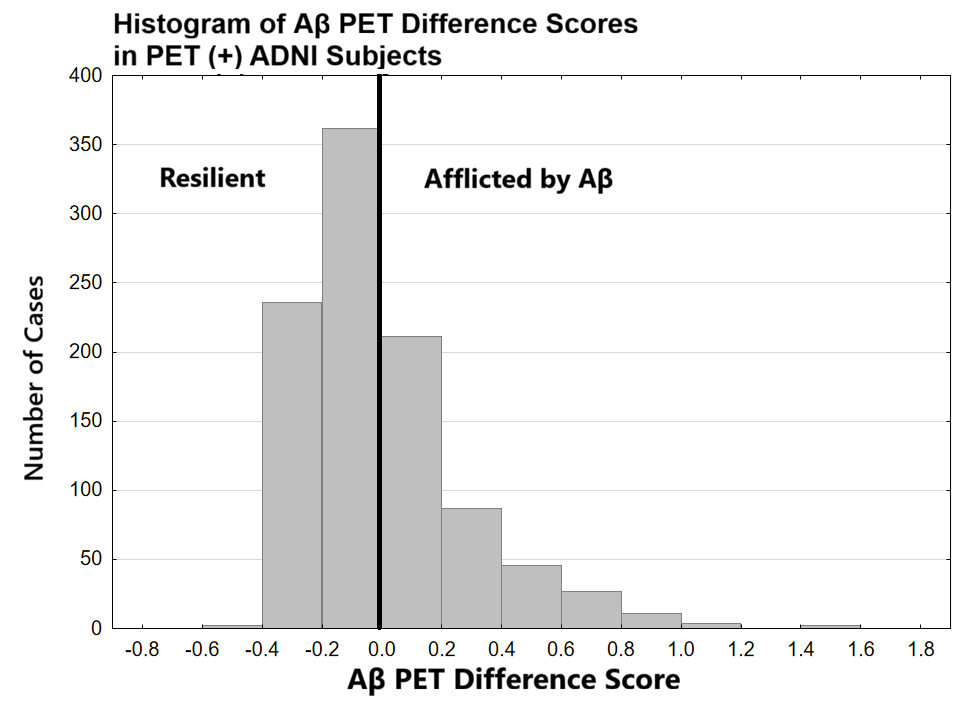


Aβ: amyloid-beta; ADNI: Alzheimer’s Disease Neuroimaging Initiative; PET: positron emission tomography
